# Development of Cloud-Based UAV Monitoring and Management System

**DOI:** 10.3390/s16111913

**Published:** 2016-11-15

**Authors:** Mason Itkin, Mihui Kim, Younghee Park

**Affiliations:** 1 Computer Engineering Department, San Jose State University, One Washington Square, San Jose, CA 95192, USA; mason.itkin@sjsu.edu; 2 Department of Computer Science & Engineering, Computer System Institute, Hankyong National University, 327 Jungang-ro, Anseong-si, Gyeonggi-do 456-749, Korea; mhkim@hknu.ac.kr

**Keywords:** unmanned aerial vehicles (UAVs), monitoring and management, collision avoidance, cloud-based application

## Abstract

Unmanned aerial vehicles (UAVs) are an emerging technology with the potential to revolutionize commercial industries and the public domain outside of the military. UAVs would be able to speed up rescue and recovery operations from natural disasters and can be used for autonomous delivery systems (e.g., Amazon Prime Air). An increase in the number of active UAV systems in dense urban areas is attributed to an influx of UAV hobbyists and commercial multi-UAV systems. As airspace for UAV flight becomes more limited, it is important to monitor and manage many UAV systems using modern collision avoidance techniques. In this paper, we propose a cloud-based web application that provides real-time flight monitoring and management for UAVs. For each connected UAV, detailed UAV sensor readings from the accelerometer, GPS sensor, ultrasonic sensor and visual position cameras are provided along with status reports from the smaller internal components of UAVs (i.e., motor and battery). The dynamic map overlay visualizes active flight paths and current UAV locations, allowing the user to monitor all aircrafts easily. Our system detects and prevents potential collisions by automatically adjusting UAV flight paths and then alerting users to the change. We develop our proposed system and demonstrate its feasibility and performances through simulation.

## 1. Introduction

Unmanned aerial vehicles (UAVs) are an emerging technology with strong implications for improving many common public and private processes. Public departments (i.e., police, public safety and transportation management) are beginning to use UAVs to deliver timely disaster warnings and improve the efficiency of rescue and recovery operations when a telecommunication infrastructure in a region is damaged or otherwise unavailable [1]. Additionally, UAVs can be used as a tool for convenience, allowing autonomous delivery systems (e.g., Amazon Prime Air [2]) to provide goods quickly to people in geographically-isolated areas. UAVs, often referred to as drones, are a type of aircraft that can fly without the need for a physical pilot onboard. UAVs are typically controlled in one of two ways: autonomously or remotely by a trained operator. The UAVs that we will be referring to are of the autonomous variety. To fly autonomously without the need for expensive visual sensors, UAVs must be constantly connected to a controlling entity, either directly or through a representative UAV, to monitor current flight status and to set an appropriate flight path.

As the density of UAVs in large urban centers increases, it becomes increasingly necessary to have a control and management system to provide an intelligent collision avoidance system [1,3]. Eventually, as UAVs are more frequently used in modern airspace, flight monitoring and collision avoidance systems will face issues of scale. Large commercial and government entities that control thousands of UAVs would want systems that can monitor all of the air traffic in real time with as little infrastructure as possible. To enable safe autonomous flight, collision avoidance must be included at the very core of monitoring solutions. These monitoring systems must be enabled to act without user approval to ensure the best possible response time and, thus, a higher probability of uninterrupted flight.

Mobility management, control and monitoring methodologies for multiple UAVs have been well studied and documented [1,3], but the implementation of such systems coupled with a comprehensive monitoring solution has not. Some UAV monitoring and control systems focus on wireless radio as the most crucial aspect of the control unit [3,4]. Other research groups have concentrated on UAV collision avoidance methods, developing complex algorithms for UAVs to avoid each other, obstacles or intruders [5,6,7,8,9,10,11]. Moreover, remote measurement and control technologies for UAVs present certain problems [12,13]. Existing solutions for UAV monitoring are difficult to deploy and are often extremely resource intensive, limiting their scalability. We aim to provide a lightweight UAV monitoring solution that can leverage the power of highly distributed cloud computing platforms to provide a UAV monitoring and collision avoidance system suitable for thousands of concurrently connected UAVs.

To pursue our goal in this paper, we develop a scalable cloud-based control and management system for UAVs, called UAV Flight Tracker. UAV Flight Tracker has a client and server model. The client allows the user to control added UAVs, receive real-time sensor updates, monitor the visualized UAVs and receive priority alerts in collision detection. The server provides sensor and collision information to the client, implements collision detection algorithms, manages user profiles and updates UAV control information. The contributions of this work are as follows:

- Design of real-time flight monitoring and visualization of multiple UAVs
- Collision detection and avoidance with automatic adjustment of UAV flight paths
- Scalability support via cloud systems
- Implementation and performance simulation

The remainder of this paper is organized as follows: Section 2 is the problem statement. Section 3 introduces existing work related to UAV monitoring and management. Section 4 proposes our UAV monitoring and management system. Section 5 explains our system in detail. Section 6 demonstrates simulation results for the operation and performance of the system. Section 7 provides concluding remarks and future work.

## 2. Problem Statement

Issues regarding various protocols for long- and short-range wireless communication between UAV systems and UAV control entities have been well researched, but solutions for a scalable monitoring system are lacking. Modern systems to support UAV communication over existing 3G and 4G cellular networks pave the way for commercial entities to deploy large fleets of autonomous UAVs in dense urban centers [12]. However, not many research projects offer a solution for a scalable monitoring and collision avoidance system that can handle a large number of concurrently connected UAVs. In addition to scalability, the ease of deployment is an important factor for modern UAV monitoring systems. As more UAVs are utilized, the number of monitoring entities will also increase, making quick, lightweight and flexible deployment an essential feature of monitoring systems.

The purpose of this paper is to address the need for a scalable UAV monitoring and collision avoidance system by providing a web application that can leverage the power of highly distributed, cloud-based systems. To properly address the problem statement, the UAV control system must provide: (1) a detailed, visually-driven graphic user interface that provides an interactive map interface and a detailed view of individual sensor data; (2) a scalable framework that can accommodate multiple concurrently connected UAV systems and with frequent sensor data updates; (3) a collision avoidance system that can send UAV flight commands and react quickly to potential collision situations; and (4) a well-designed framework that can be deployed, cleared and redeployed easily on cloud-based computing services.

## 3. Related Work

Though the concept of UAVs is promising as an emerging technology, the field is relatively new to industry and is much less developed than other military applications. There are various issues to address in order to realize the effective, stable and reliable use of UAVs, i.e., network topology, routing, seamless handover, energy efficiency and management [1]. As the number of potential users of UAVs increases, it is especially important to provide mobility management, control and monitoring including collision avoidance of intruders or obstacles [8,10].

Some UAV monitoring and control systems focus on wireless radio as the most crucial aspect of the control unit. In a centrally-designed system, the controller radio facilitates communication between every UAV in the system; without the controller radio, the system could not function. Lam, T.M.; et al. [3] consider a radio delay to control UAVs. Tele-operation of a UAV may involve time delay due to signal transmission. This would result in poor operator performance and control difficulties. Thus, the authors describe a theoretical analysis using wave variables with a collision avoidance system for UAV tele-operation with time delay. Kim, H.N.; et al. unburden the controller radio by allowing every UAV in a particular swarm to connect to and manage one another [4]. To manage this type of peer-to-peer control over several UAVs, a comprehensive methodology is needed to replace nodes seamlessly in the swarm. This is accomplished by creating a second revised routing table sent to the swarm and saving it in the background until the new UAV is connected. Once the new UAV is connected, the new routing table is used by the swarm, and the process to optimize this table begins. The obvious advantage to the system described in [4] is that there is no central communication point; thus, an outage of the ground control system or controller radios would not cause a system-wide failure. However, restricting UAV control to the swarm means that all UAVs must start within a specific range of one another, making routing complicated. Additionally, distance constraints on UAVs limit the full utilization of the system, as it cannot track UAVs that are flying long distances in opposite directions. Further, scaling of this system can be expensive and ultimately impractical with the routing process becoming more and more complex as the number of concurrently connected UAVs grows. Eventually, the routing process could exhaust the computational power of the UAVs, and all UAVs in the system would need to be upgraded. It is also important to note that in an ideal system, only small computations are performed on the UAV. This is preferred as onboard computations quickly increase UAV hardware costs and flight time by draining battery power.

Many research groups attempting to develop innovative ways to control UAVs have concentrated on collision avoidance, anticipating a large number of UAVs, obstacles or intruders. The paper by Kuriki, Y.; et al. [5] presents a cooperative control strategy for collision avoidance in a multi-UAV system by using decentralized predictive and consensus-based control. Liu, W.; et al. develop a novel path planner to control multiple UAVs synchronously based on distributed path generation and a bi-level programming technique. Patel, A.; et al. propose an autonomous collision avoidance system using an acceleration-matching algorithm to increase autonomy in connected UAVs [7]. The paper by Ling, L.; et al. utilizes a particle filter to estimate the target state accurately for collision avoidance [8]. The paper by Alejo, D.; et al. [9] presents a collision avoidance method for multiple UAVs and other non-cooperative aircraft based on velocity planning and trajectory prediction of a particle filter. Yoo, C.S.; et al. implement a collision detection and avoidance algorithm on a digital flight control computer [10]. Sharon Lozano at NASA has implemented a cloud-based Unmanned Aircraft Traffic Management (UTM) system that provides a way for civilian pilots to reserve airspace [14]. This system maintains a database of reserved and active flights, providing information to pilots about adverse weather conditions and restricted airspace. The UTM project consists of four technical capability phases, ultimately enabling the management of UAVs in high-density urban areas with large-scale contingency mitigation. The paper by Zhu, L.; et al. [12] assumes UAVs may be operating in joint missions with manned aircraft, e.g., helicopters, and use the concept of automatic dependent surveillance broadcasting to collect aircraft data used in collision avoidance systems. Based on flight maneuvering, the proposed detection algorithm creates a sector range to cover possible flight direction changes of UAVs and helicopters.

Moreover, remote measurement and control technology for UAVs are vital, leading Ling, L.; et al. [8] to build an onboard embedded computing platform that realizes the remote measurement and control of the UAV. Their solution utilizes a UAV position compensation method based on network time delay prediction. This remote measurement platform increases the accuracy of control and management, but fails to consider collision avoidance and provides no mention of performance when connecting a large number of UAVs. The paper by Roldán, J.J.; et al. [13] focuses on using a central control system to move several mini-UAVs around a greenhouse, gathering sensor information. The employed system has only a central service that performs all of the sensor computations, manages flight paths and controls external actuators for the greenhouse operation. The advantage of this system is that a centralized control point controls all UAVs and performs all resource intensive computations. This results in each UAV being inexpensive, but at greater risk, as a failure of the central control point would cause the entire system to fail. Additionally, relying on a central control radio either limits the UAVs to a relatively short distance from the controller or drives up the cost of the radio.

While those solutions explained earlier [3,4,5,6,7,8,9,10,11,12,13] may satisfy some of the use cases presented, they do not fully address the need for a truly scalable UAV control system. The aforementioned solutions present no way to deploy the monitoring system easily, nor do they mention the use of a highly distributed system, a key component of UAV Flight Tracker. Most importantly, the related papers did not thoroughly test their UAV control systems to prove that their solutions are reliable on a large scale.

## 4. Architecture of the UAV Monitoring and Management System (UAV Flight Tracker)

This section presents our cloud-based UAV monitoring and management system, called UAV Flight Tracker. The novelty of our system is to allow monitoring of real-time UAV sensor data, a visual display for current flight paths for multiple concurrent UAV flights and support for automatic collision avoidance. The UAV Flight Tracker architecture consists of three entities: the client, server and UAV. Normal operation of our system utilizes a single cloud-hosted server with a varying number of clients and UAVs.

**Figure 1:**
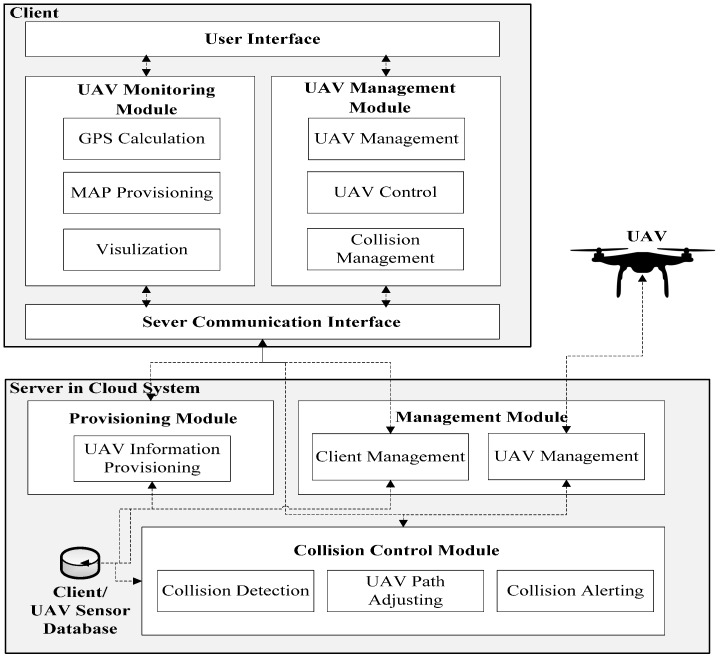
System Architecture of UAV Flight Tracker.

The client includes UAV monitoring and UAV management modules that allow the user to control added UAVs and receive real-time sensor updates, as well as user and server interfaces with which to communicate, as shown in Figure 1.

The UAV monitoring module allows users to receive real-time sensor information and visually monitor their UAVs using a 2D mapping interface. Data from the server communication interface of the client system is passed to the UAV monitoring module for processing before being sent to the map and displayed on the user interface. The map uses GPS calculations, map provisioning and other visualization services to display the current UAV location, UAV destination and a direct flight path.

The UAV management module allows the user to add any number of UAVs to the server and send live path data, utilizing three sub-modules: UAV management, UAV control and collision management. The sub-module for UAV management allows the application to associate UAVs with users and grant access to monitoring and control features. The UAV control sub-module is responsible for sending the live path data to the server, where it can be deployed to UAVs. The collision management sub-module provides a way for the user to set preferences for automatic path redirection and receive priority alerts from the collision detection algorithm.

The server of UAV Flight Tracker provides a provisioning module, a management module, a collision control module and a database. Together, these modules serve sensor and collision information to the client system, implement the collision detection algorithm, manage user profiles and update UAV control information.

The server provisioning module facilitates communication of sensor data between the databases of server and client. The UAV information provisioning sub-module defines application program interface (API) routes to the database. This allows simple requests to perform create, read, update and delete (CRUD) operations on sensor datasets in the database.

The management module keeps track of the clients and associated UAVs that are currently being utilized in the application. The client management sub-module controls the association between a specific client instance and flight control permissions for the connected UAV. The UAV management sub-module tracks the connected UAVs and sends authorized commands in real-time to deployed UAVs.

The collision control module implements the collision control algorithm, performs necessary UAV path adjustments and alerts the client to potential collisions and UAV path changes. The collision detection sub-module implements the collision detection algorithm by reading real-time sensor information from the database, checking for collisions and then preparing data to make the needed UAV path adjustments. The UAV path-adjusting sub-module receives collision information from the collision detection sub-module, performs the path adjustments and sends the updated path to the management module where it can be deployed to the UAV. The collision alerting sub-module reports detected collisions and adjusted path data to the collision management module of the client, where it can then be displayed to the user.

**Figure 2:**
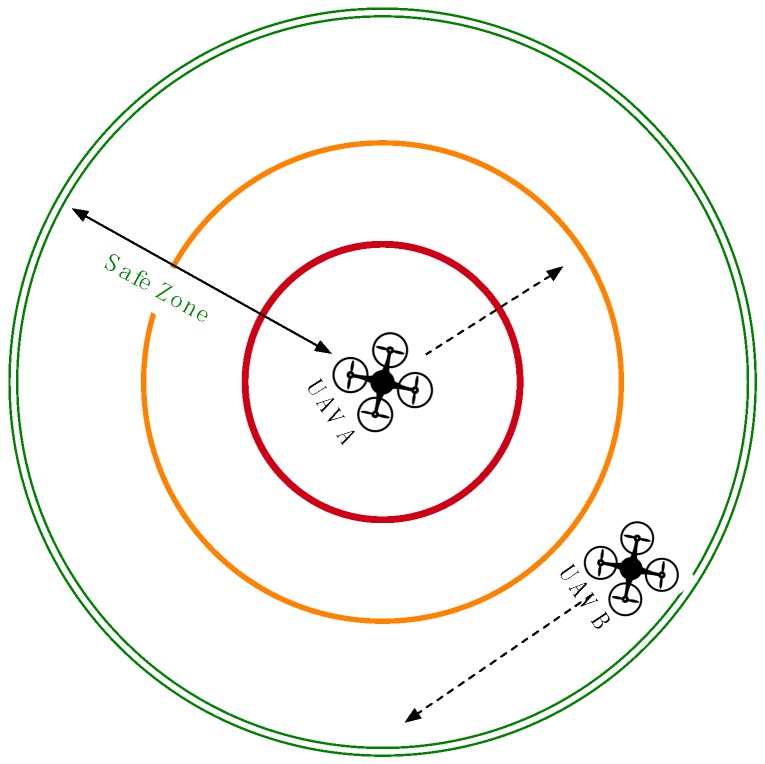
Case 1: closely monitor UAV B in the safe zone.

**Figure 3:**
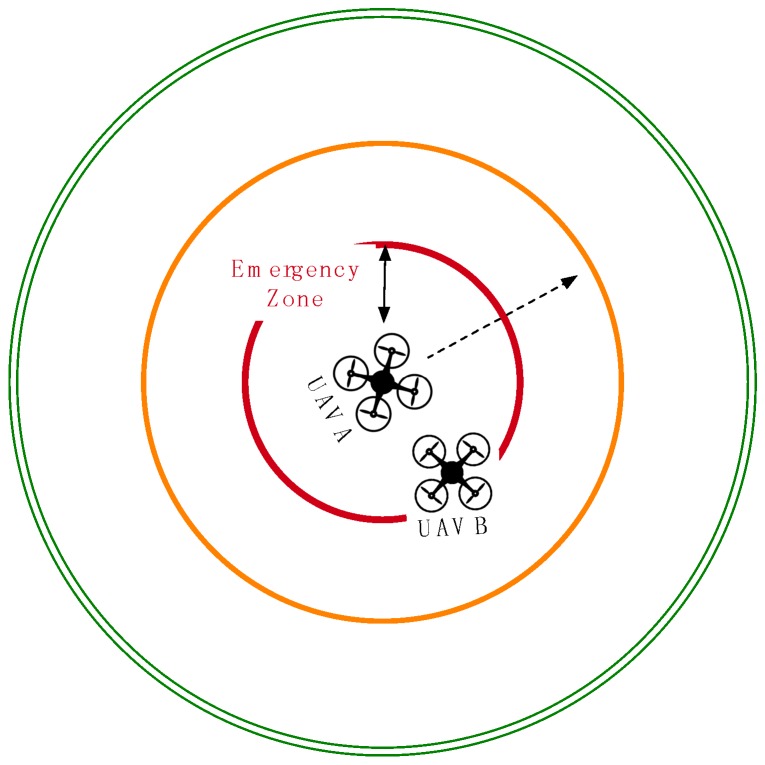
Case 2: stop movement for UAV B in the emergency zone.

To design the collision detection algorithm on the server, we assume that each UAV has a safe zone, a redirection zone and an emergency zone, as shown in Figure 2 and Figure 3. Of these three zones, the safe zone has the biggest radius around each UAV, e.g., 1000 m, and is used to designate a completely safe flying distance for adjacent UAVs. All of the connected UAVs that are flying within the safe zone of another UAV are flagged as being adjacent and are checked at frequent intervals. The redirection zone has a middle radius around each UAV, e.g., 500 m, meant to indicate the distance at which adjacent flying UAVs should be redirected. UAV redirection is an adjustment of the connected UAV heading as needed to redirect the UAV away from the UAV or UAVs whose redirection or emergency zone it has entered. This new temporary heading will be held until the offending UAV moves out of the redirection zone. UAV redirection occurs with the UAV moving at the slowest speed. If all UAVs are traveling at the same speed, then one of the violating UAVs can be selected with a specific criterion (e.g., randomly, lower ID or more energy) to have its course redirected. The emergency zone has the smallest radius around each UAV, e.g., 300 m, where it is determined that no UAV should be flying. When any UAV is found to be within the emergency zone of another UAV, both aircrafts will be immediately stopped mid-flight before being redirected, thus preventing a potential collision. The radii of the three zones can be altered according to the average speed, wingspan and movement accuracy of the monitored UAVs. Algorithm 1 shows the pseudo code for the collision avoidance.

**Algorithm 1: Collision Avoidance**1.  void CollisionAvoidance(UAV[i], UAV[j]) { 2.   // **Input:** UAV[i], UAV[j] 3.   // **Case 1:** Monitor closely for changes in flight path 4.   if UAV[i] is within UAV[j].safeZone{ 5.   UAV[i].setAdjacent(UAV[j]); 6.   7.   // **Case 2:** UAV in emergency zone 8.   if UAV[i] is within UAV[j].emergencyZone { 9.   Stop UAV[i] for 10 s; 10.  RedirectHeading(UAV[i],UAV[j]); 11.  } 12.  // **Case 3:** UAV in redirection zone 13.  else if UAV[i] is within UAV[j].redirectionZone{ 14.  RedirectHeading(UAV[i],UAV[j]); 15.  } 16.  } 17. }

In Case 1 of Figure 2, UAV B is flying within the safe zone of UAV A, causing UAV B to be monitored at a more frequent interval by the collision avoidance module. These more frequent updates ensure that redirection can take place quickly if UAV B moves into the redirection zone of UAV A.

In Case 2 of Figure 3, UAV B has entered the emergency zone of UAV A and is prevented from moving for 10 s in order to prevent immediate collision. The stop time can be raised according to the average speed of the UAVs. The collision avoidance module sends a signal to the management module of the server on UAV Flight Tracker in order to stop the movement of UAV B and keep the aircraft hovering in place.

**Figure 4:**
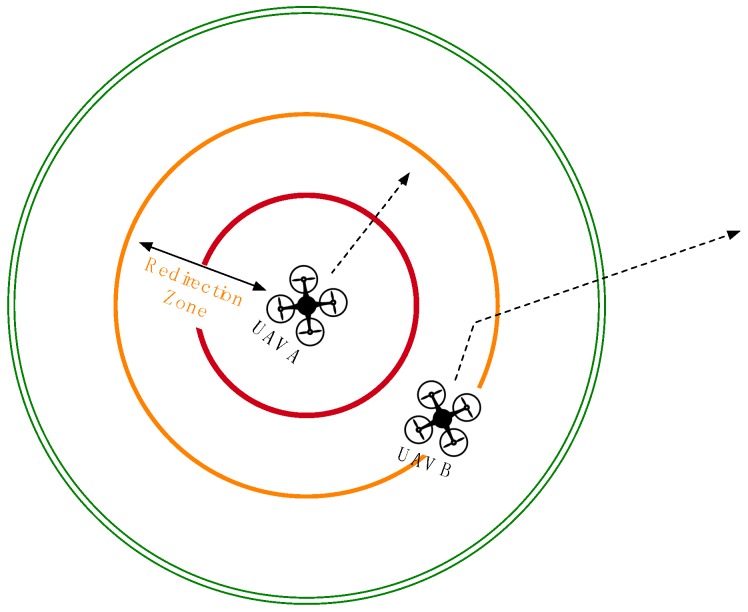
Case 3: change flight path of UAV in the redirection zone.

In Case 3 of Figure 4, UAV B has entered the redirection zone of UAV A and has its flight path redirected to avoid any potential collision with UAV A. The collision avoidance module informs the management module to redirect UAV B so that UAV B does not enter the emergency zone of UAV A.

## 5. Development of the UAV Flight Tracker

This section details the development of our proposed UAV Flight Tracker by discussing the general web application packages utilized by the system.

### 5.1. Sketch for Development of UAV Flight Tracker

**Figure 5:**
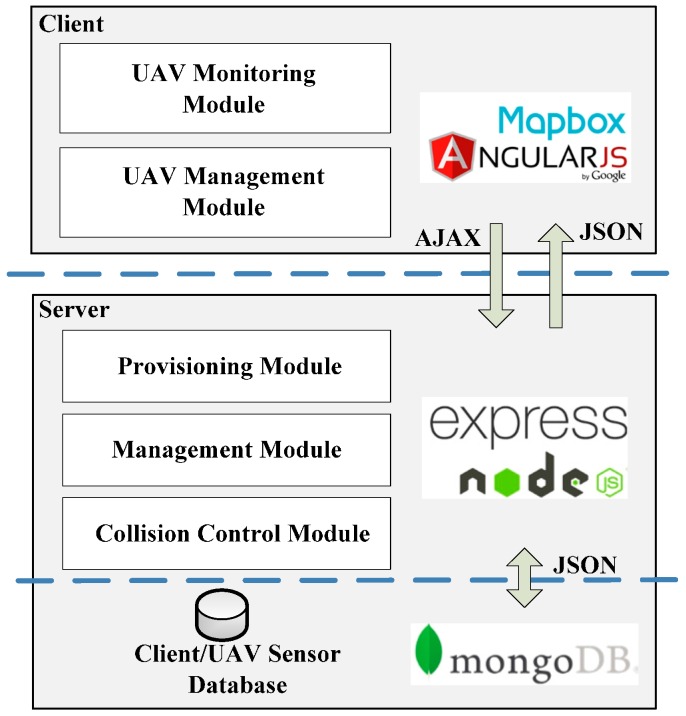
Development design of UAV Flight Tracker.

UAV Flight Tracker is built on the MEAN stack [15] using MongoDB [16], ExpressJS [17], AngularJS [18] and NodeJS [19], as shown in Figure 5. The provisioning module of the server provides HTTP connection listening, routes for the API endpoints and a constant connection with the MongoDB database to read and write JSON documents [20]. The management module of the server manages active client sessions and connected UAVs, controlling communication privileges between the two. The collision control module implements the collision avoidance algorithm by reading sensor data from the database to determine UAV locations and sending the appropriate commands for UAV redirection (i.e., direction and speed) to the management module. Additionally, collision avoidance reports and UAV redirection information are sent to the provisioning module where it can be presented to the user. NodeJS and the Express framework are especially well suited to a real-time UAV tracking application because they function using non-blocking I/O calls, providing low response times for multiple concurrent connections; this is a key factor that allows UAV Flight Tracker to easily scale up.

The client system is implemented as an AngularJS web application that provides real-time UAV sensor monitoring and interactive location mapping. The client’s UAV monitoring module uses Angular directives to manipulate the webpage document object model (DOM) [21] to display the sensor data retrieved from the server. Data in the DOM are displayed and formatted on the HTML page or used to draw objects on the map. The client uses Leaflet [22], an interactive JavaScript map library, to plot UAV coordinates, paths and destination markers. A community library for Leaflet Directives [23] is used, as Leaflet does not natively support Angular Directives. The client uses asynchronous JavaScript and XML (AJAX) for HTTP requests. AJAX is utilized as part of the AngularJS client application by providing a way for the client to make asynchronous HTTP requests to the server.

### 5.2. Implementation of UAV Flight Tracker

The implementation of the client and server of UAV Flight Tracker can be split into three main files. The server is composed of a single JavaScript file (server.js) running on NodeJS, while the client is composed of two files, the main application JavaScript file (app.js), written with AngularJS, and an HTML file (map-view.html).

**Figure 6:**
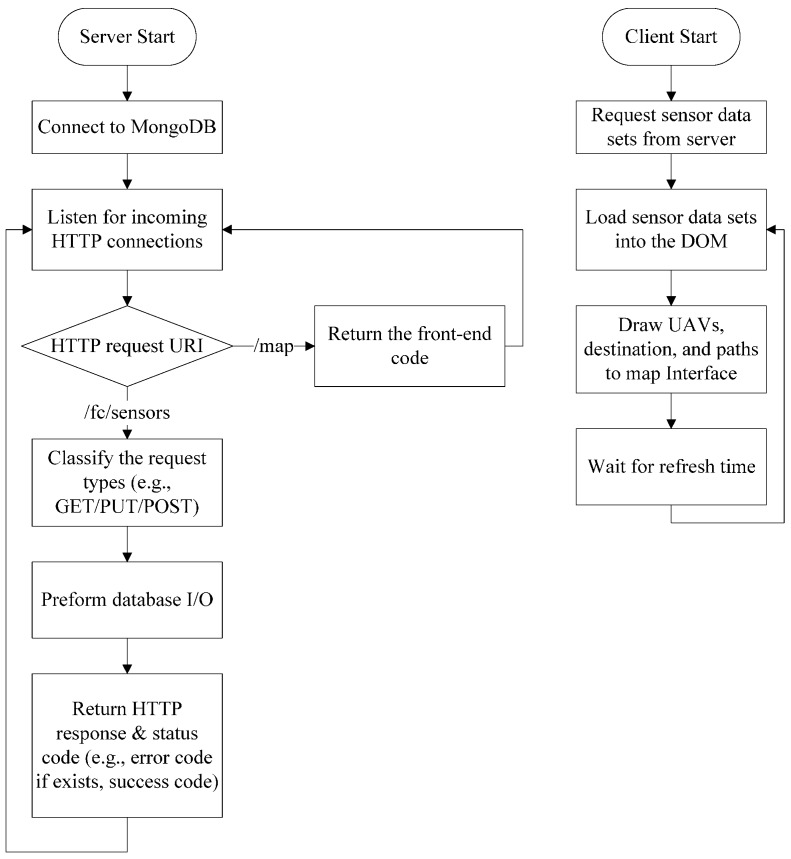
Flowchart for server and client.

The server.js file hosts the frontend application, manages the MongoDB database connection and provides API routes for the sensor data. UAV Flight Tracker uses the Express framework to aid in the construction of the NodeJS server by drastically reducing the code complexity of the server. The first task for the server as it first runs is to connect to the MongoDB database. Connection to the database is done with a call to the MongoDB uniform resource identifier (URI), found in the environment file, which will return a database object. After securing communication with the database, the server is initialized and is ready to identify incoming connections over the port specified in the environment file. HTTP GET requests that the ‘/map’ URI return the frontend code (i.e., HTML, CSS and JavaScript), while all requests to the ‘/fc/sensors’ URI are routed to the API endpoint where the specific request (i.e., GET, PUT and POST) can be handled. Any errors the server encounters when looking up a specific URI are caught by a generic error handler and the appropriate error code or a generic HTTP 500 error is returned. All of the routes to the ‘/fc/sensors’ endpoint, including GET, PUT and POST, are all defined in the ‘server.js’ file as separate functions. In each of these functions, the specific type of HTTP request along with the request data are passed to the function body, where it is used to perform the specific request. In the example of an HTTP GET request, the requested sensor dataset is searched for by unique id in the database, returned to the server and then sent back to the requestor. Similarly, in the example of an HTTP POST request, the data to be written are parsed by the server from the POST request body to the database, and then, a successful status code is sent to the requestor. The Figure 6 shows the flowchart for the operation of the server and client.

On the client-side, the app.js file dictates the behavior of the front-end of the application by using a route provider, a set of services for API requests and a JavaScript controller definition for the main mapping interface. The route provider defines the content template URL, service resolutions and the JavaScript controller for a specific URI of the web application. The content template is the HTML content page that is loaded inside of the main HTML template when a page is selected. By modeling the front end in this way, multiple monitoring pages can be displayed without having to duplicate any HTML code. Service resolutions, in this application, are the sensor datasets pulled from the API that the JavaScript controller needs in order to function properly; the resolutions occur before the JavaScript controller runs. The JavaScript controller, in this case “MapBoxController”, is selected by the route provider to be paired with the html template.

All of the map rendering, GPS coordinate calculations and sensor formatting are done in the “MapBoxController”. When run, the JavaScript controller initializes all of the variables used by the Leaflet mapping interface, including the map view location, all displayed markers and all drawn paths. Next, a call to the “/fc/sensors” endpoint is made using a service in the “Sensors” services set to pull and update the map display information that has already been initialized. It is important to note that communication between the JavaScript controller and the API is defined according to the functions of the service set. These functions formulate GET and PUT requests to the API endpoints that are provided by the NodeJS server. Inside the callback from the sensors’ GET request, data for the latitude and longitude of all UAVs and paths pass to the map directive to be drawn.

**Figure 7:**
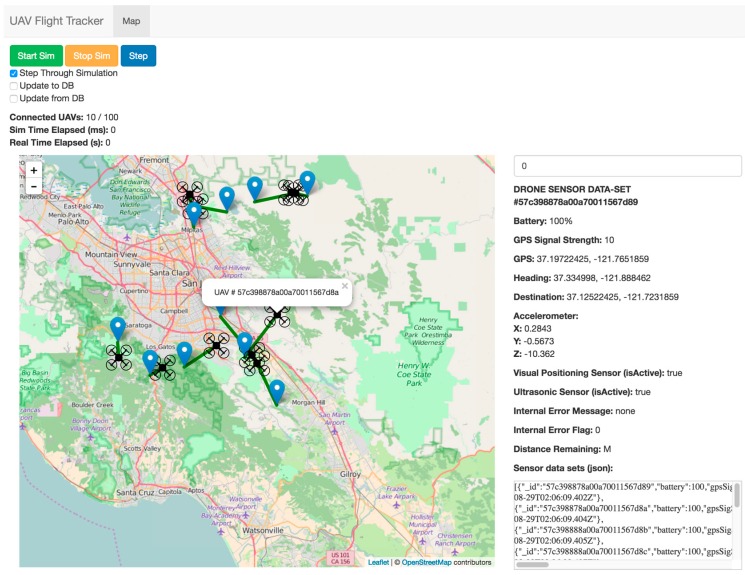
A screen on the client system.

Figure 7 shows the main screen used to visualize UAVs from sensor data. This client graphic user interface (GUI) consists of a top bar filled with three buttons, a main map interface on the left-hand side and a detailed data view on the right-hand side. The top bar of the client GUI provides three buttons for the user to start a simulation based on the UAV sensor data that exist in the database. During the simulation, UAVs move along their simulation path, and all aspects of UAV Flight Tracker operate normally. Options are available for the simulation to update local sensor values from the database or update all sensor datasets to the database on an interval of 100 ms. The main map interface shows connected UAV locations, set destinations and paths. The user can click on any of the map items to see the id (identity) of the UAV to which a particular piece of data refers. On the right-hand side of the GUI, a listing for all information from the sensor data variables being tracked can be viewed in real time. An input box for the currently selected UAV at the top of the pane allows the user to change the UAV sensor dataset being viewed. 

**Figure 8:**
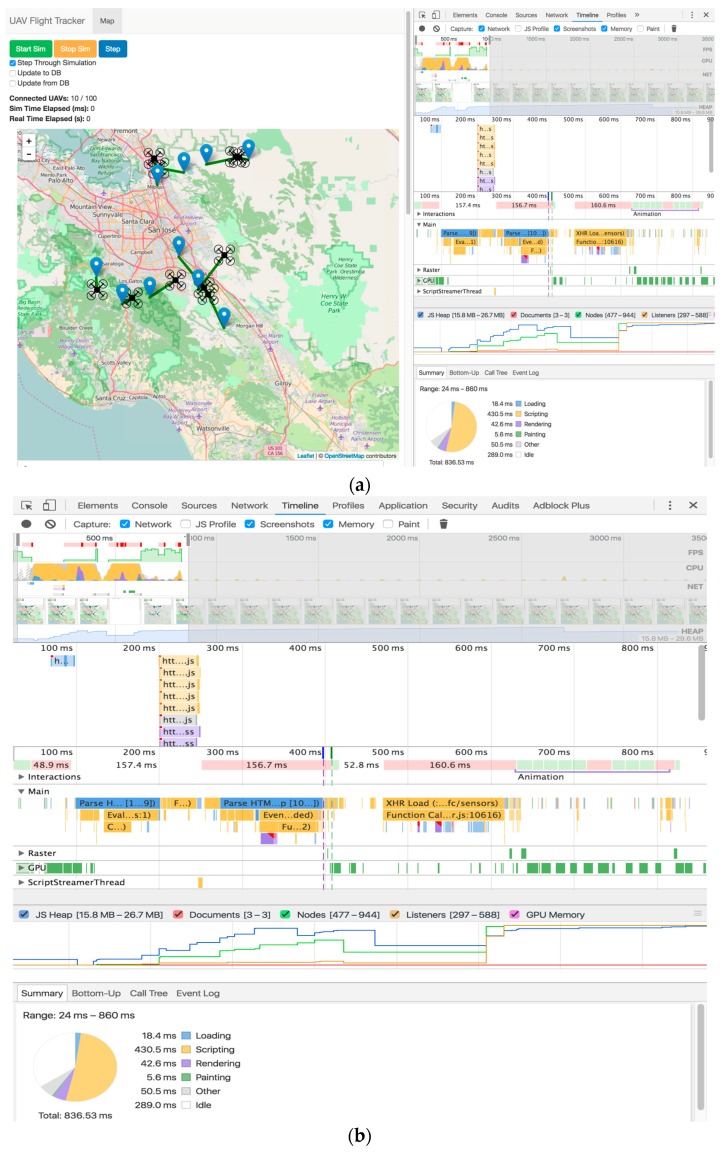
Client performance testing results by Chrome DevTools: (**a**) screen of client system with performance results and (**b**) performance results.

Figure 8 shows the performance results provided by Chrome DevTools [24] while we were simulating the flight of UAVs. We can see the mobility of UAVs together with the variance in performance as shown in Figure 8a. The performance timeline in Figure 8b details nearly all of the technical information gathered from the server and client performance testing. From this performance-monitoring tool, the maximum and minimum values for client memory utilization and a timeline with the timing of all round trips for all requests sent from the client to the server could be easily monitored.

## 6. Performance Evaluation

We implement UAV Flight Tracker and evaluate the performance in terms of response time, storage/memory overhead and collision avoidance.

### 6.1. Setup and Testing Methodology

UAV Flight Tracker runs on Heroku [25], a cloud application platform that provides a NodeJS runtime and MongoDB service. A JSON package file lists the versions of NodeJS (Version 4.4.7), Express (Version 4.13.3) and MongoDB (Version 2.1.6) that will run on the cloud service once deployed. Heroku automatically manages all server provisioning and redundancies. On the client, minified versions of Jquery (Version 2.1.4), Bootstrap (Version 3.3.4) and AngularJS (Version 1.4.6) are used along with Leaflet (Version 0.7.7) and Angular Leaflet Directives. The client machine used for development and testing has an Intel Core i7 @ 2.3 GHz with 16 GB of DDR3 memory clocked at 1600 MHz.

Testing of UAV Flight Tracker is conducted with the server and database hosted on Heroku and a laptop (with specifications in the setup description) to run the client. All tests in the performance evaluation are conducted with the same test set of 100 UAV sensor datasets. Additionally, all subsets of the initial 100 datasets are also identical, meaning that a subset of 20 datasets used in one test is identical to the subset of 20 datasets used in another test. Each sensor dataset used for testing contained unique, randomized GPS coordinates for the UAV start location and the destination. A random point generator provided by the ‘Geographic Midpoint Calculator’ website [26] is used to generate the initial GPS coordinates for UAVs. Coordinates are generated around a center point in San Jose, CA, USA, and a maximum distance of 25 km. For each individual set of coordinates, a unique destination 5 to 14 km from the UAV GPS location was randomly generated using the equations set forth as Equations (1) and (2). The variable *gpsCoordinate* is the UAV starting location, and the *destinationCoordinate* is the resulting randomized destination through a random function with a range between *a* and *b*, RANDBETWEEN (*a*, *b*). Equation (1) uses the GPS latitude to calculate a randomized destination latitude, and Equation (2) uses GPS longitude to calculate a randomized destination longitude.

*destinationCoordinateN* = *gpsCoordinateN* + RANDBETWEEN (−0.009, 0.009) (1)

*destinationCoordinateW* = *gpsCoordinateW* + RANDBETWEEN (−0.009, 0.009) (2)

To express the performance and scalability of UAV Flight Tracker adequately, the latency of data sent between the client, server and UAV must be recorded along with the size of data being held on the server. This means that the roundtrip request latency must be measured for communication between the client and server, as well as between the UAV and server. Additionally, the memory utilization of the client is monitored to gauge front-end performance and ensure that the UAV Flight Tracker client runs on portable machines with fewer available resources. The number of concurrently active UAVs is the most significant factor in determining server response time and client performance. Therefore, the number of connected UAVs is used as the domain over which we gauge HTTP request time, required client memory size and database size on the server. We increase the number of UAVs from one UAV to 100 UAVs.

We then measure the effects for collision avoidance as follows:

- Server performance: response times in PUT, GET and POST operations
- Overhead: required database size on the server and required memory size on the client
- Collision avoidance: total UAV collisions and UAV mission completion time

All HTTP request times and required client memory information are provided using Google Chrome DevTools. The MongoDB size is provided by the web interface for mLab Database Service [27]. For testing of collision avoidance, we set up 300 m, 500 m and 1000 m as the radii of the safe, redirection and emergency zones, respectively.

### 6.2. Results Analysis

#### 6.2.1. Results on Server Performance

(1) Sensor data update time (PUT) vs. connected UAVs

Sensor data update time is the total time that the client needs to update every sensor dataset in a series. For every connected UAV, a single HTTP PUT request is sent to the server. Each request is sent in a series, meaning that the total roundtrip time of all PUT requests should scale linearly with the number of connected UAVs. The total roundtrip time is the total time needed to send all of the requests, update them in the database and receive back a response from the server. The total sensor data update time starts with the exact time that the first PUT request is sent and ends when the server response from the last PUT request is received. Total time is a good indicator of sensor update performance and system performance in general, because the client sends all PUT requests in a series, one after the other.

**Figure 9:**
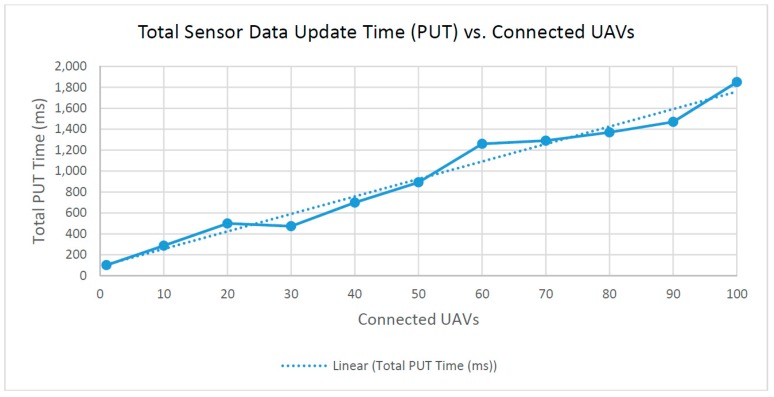
Total time to complete sensor data update.

As shown in Figure 9 the time taken to update all of the connected UAV sensor datasets is linearly proportional to the number of connected UAVs. The fitted linear approximation of the recorded data has an R-squared value of R^2^ = 0.97417; in statistics, R-squared is the coefficient of determination, i.e., the number that indicates the proportion of the variance in the dependent variable that is predictable from the independent variable [28]. This is expected as each additional connected UAV provides another dataset to be updated via an HTTP PUT request to the server.

(2) Total sensor retrieval time (GET) vs. connected UAVs

The sensor retrieval time is the roundtrip time for a single HTTP GET request from the client to reach the server, query the database and return with the requested sensor datasets. This total is used to describe the sensor retrieval time because all of the UAV sensor datasets are being retrieved, though only one HTTP GET request is sent from the client. Unlike the previous test for total sensor update time, where a series of HTTP PUT requests are being sent, this test requires only a single HTTP GET request to retrieve all sensor datasets.

**Figure 10:**
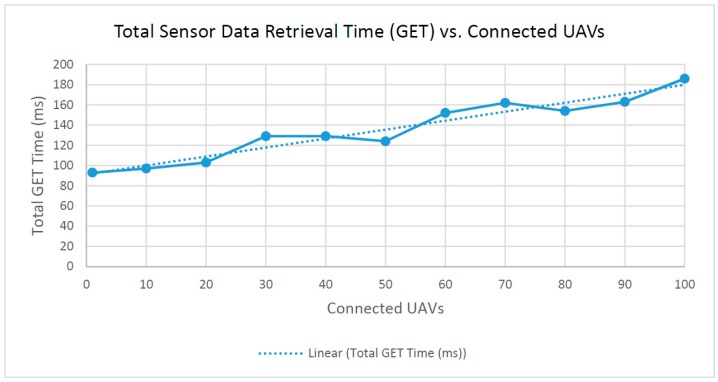
Total time to complete sensor data retrieval.

As shown in Figure 10, the fitted linear approximation of this graph has an R-squared value of R^2^ = 0.93346. This linearly increasing trend is expected, because the database query time and the time needed to send the server response increase as the dataset becomes larger with more connected UAVs.

(3) Average sensor data insertion time (POST) vs. connected UAVs

**Figure 11:**
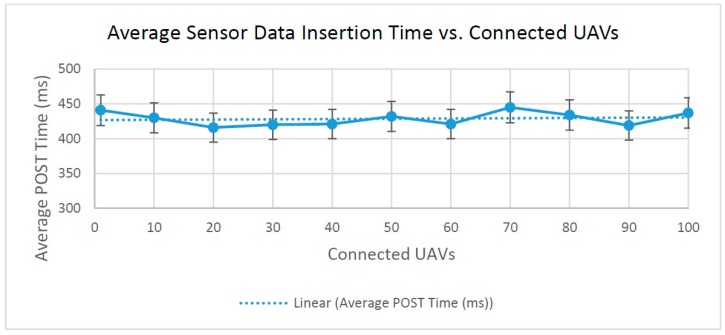
Average time for sensor data insertion.

The sensor data insertion time is the roundtrip time for an HTTP POST request issued by a newly connected UAV to add its sensor data to the database for the first time and return an HTTP code to the requesting entity. After receiving the request, the server copies the new JSON data to the database, and a unique ID and timestamp are then associated with the document. The time taken to perform an HTTP POST request to the server should not depend on the current number of connected UAVs, as the database is able to add JSON documents without modifying existing data. In a production environment, sensor data insertion requests are likely to be spread throughout a large interval of time with the occasional cluster of new UAVs connected to the system. As a result, the test for sensor data insertion time is calculated as the average of requested roundtrip times to display performance in a more realistic scenario. As expected, Figure 11 shows similar values even as the number of UAVs increases. Though the results vary a bit, it is important to note that all of the measured insertion times are within 5% of 426.83 ms.

Server performance testing highlights a few perceived inconsistencies in the roundtrip times for HTTP requests. When scaling the number of connected UAVs, HTTP PUT requests for updating all of the sensor datasets prove to be the costliest operation, taking 1850 ms to update 100 connected UAVs. Compared to the HTTP GET request’s high of only 186 ms at 100 connected UAVs, processing PUT requests to update sensor data seems abnormally high. However, this abnormality is explained by considering the number of HTTP requests needed to insert, update or retrieve data from the server. Sensor data retrieval (HTTP GET) requests use an array of JSON sensor data sent within the body of a single request, while individual sensor update (HTTP PUT) requests are sent for each sensor set that needs to be updated. Therefore, the added latency of PUT requests is not unexpected, but could be avoided in the future if PUT requests were sent in parallel.

#### 6.2.2. Results on Overhead

(1) Required server database size vs. connected UAVs

**Figure 12:**
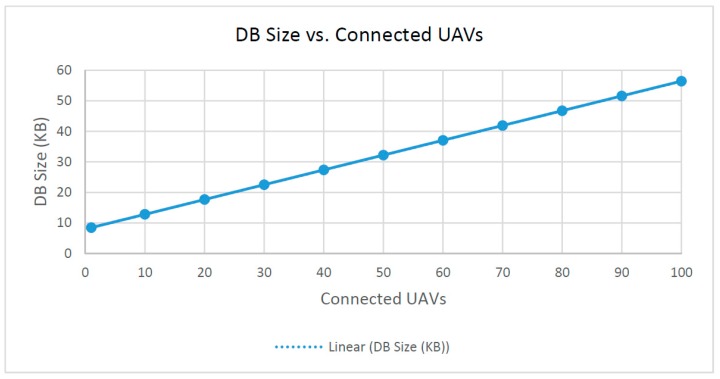
Required DB size on server vs. connected UAVs.

The database size is the total size of the MongoDB that holds all sensor datasets in individual JSON documents. As shown in Figure 12, the size of the DB collection in kilobytes is linearly proportional to the number of connected UAVs. This result shows that each UAV has the same number of key and value pairs held in its respective sensor dataset. The fitted linear approximation of this graph has a perfect R-squared value of R^2^ = 1.

(2) Required client memory size vs. connected UAVs

**Figure 13:**
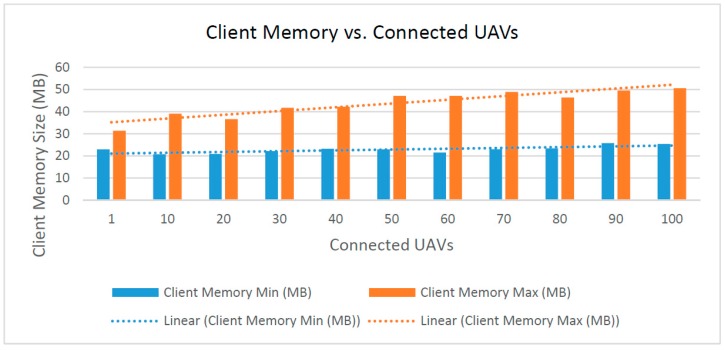
Required memory size on client vs. connected UAVs.

The client memory size is the minimum and maximum client system memory used by the UAV Flight Tracker during normal operation. The maximum consumed memory trends higher as the number of connected UAVs increases, while the minimum or baseline memory stays relatively static, as shown in Figure 13. The fitted linear approximation of the maximum client memory trend line has an R-squared value of R^2^ = 0.85785. Most of the client memory is used to draw the map interface and maintain a list of sensor data objects. Thus, as more UAVs and destinations are added to the map, the client uses more memory. When the client interface initially loads, there are no objects drawn to the map until the server fulfills the HTTP GET request for sensor data. Therefore, the baseline memory usage does not depend on the number of connected UAVS and instead stays consistent.

#### 6.2.3. Results on Collision Avoidance

(1) Total UAV collisions (without collision avoidance)

**Figure 14:**
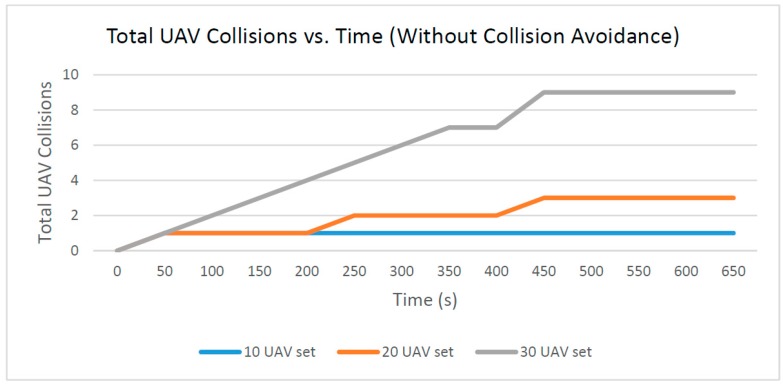
Total UAV collisions.

The total number of UAV collisions that occur over time is a measurement of how many active UAVs are within another UAV emergency zone at any given time during their respective flight paths. Any given UAV can only crash once, so any subsequent crashes reported for that UAV will be ignored as erroneous in this dataset. Every UAV path is linear, spanning from a randomly-generated origin to a randomly-generated destination according to Equations (1) and (2). As shown in Figure 14, the number of actively connected UAVs increases the likelihood of collisions occurring.

(2) UAV mission completion time (with/without collision avoidance)

**Figure 15:**
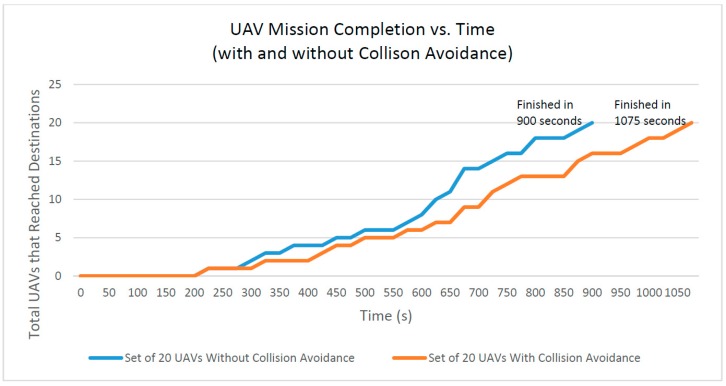
UAV mission completion time.

UAV mission completion is the total number of connected UAVs that have completed their missions by reaching their respective destinations. In the experiments, all UAVs move at the same speed and their path distances change depending on the randomly-generated origin and destination points. This test is repeated twice with or without the collision avoidance. In other words, we enable the collision avoidance to redirect active UAVs while disabling the collision avoidance system, rendering it unable to change UAV flight paths. As a result, Figure 15 shows that the finishing time that the set of UAVs without collision avoidance reach a target or a destination is quicker than one for the set of UAVs with collision avoidance. This is the reason that some UAVs would collide and never reach their destination. Redirection in the collision avoidance test occurs as the collision avoidance algorithm description detailed in the Section 4. It normally adds anywhere from two seconds to one minute of extra flight time per incident. In Figure 15, with a set of 20 connected UAVs, the normal, uninfluenced time of completion is 900 s, while the time of completion for redirected flight is 1075 s. This test is based off of the same sample flight data used in the previous test for total UAV collisions. This result shows that the redirection algorithm only adds a total of 175 s to the overall completion time.

## 7. Conclusions

Developing a UAV monitoring and collision avoidance system that is both scalable and simple to deploy is a challenging task. This paper has proposed a UAV flight tracking solution that allocates critical and non-critical computing intensive processes between the server and the client, allowing for optimal performance on a large scale. The server handles critical processes for collision avoidance, UAV control and sensor updates. Following this distribution, more trivial tasks (i.e., map rendering and sensor display) are left for the client to compute. This allows the server to run on a distributed cloud-hosting platform, ensuring that the most important aspects of UAV tracking run with a scalable resource pool.

As proposed in [8], the future of commercial and other non-military UAV flights will use 3G and 4G cellular networks to facilitate UAV communication with the controlling entities. Our work aligns well with this proposal by providing a cloud-based web application that monitors and controls UAVs over the HTTP protocol. As UAVs are easily connected to cellular networks, providing them with nearly limitless flight possibilities, our flight monitoring solution matches that flexibility by allowing for easy deployment of the entire system on cloud-based computing services. This flexibility continues from the server deployment to the client, as the user-facing application can run in any modern web browser and requires a small amount of system memory.

Future work on this paper will continue to provide collision detection in three dimensions, improve the performance of the client application and conduct tests with non-simulated UAVs. We will optimize the radii of the three zones (i.e., safe zone, redirection zone and emergency zone) to enhance the performance of UAVs, i.e., in terms of mission completion time. Moreover, collision detection accuracy depends on the specifications of the UAV system utilized. The accuracy of the GPS signal and average radio delay affect the radii of collision avoidance zones needed to ensure safe flight. Continued research on the specific attributes of UAVs that determine safe flight distances would complement our UAV management system design.
